# Interpretable machine learning for low-sample multi-omics: a case study of ferret vaccine response

**DOI:** 10.1093/bioadv/vbag167

**Published:** 2026-06-12

**Authors:** Nehleh Kargarfard, Robert Dunne, Carol Lee, Laurence Wilson, Alexander J McAuley

**Affiliations:** Australian e-Health Research Centre, Commonwealth Scientific and Industrial Research Organisation, Sydney, NSW 2145, Australia; Data61, Commonwealth Scientific and Industrial Research Organisation, Sydney, NSW 2015, Australia; Australian e-Health Research Centre, Commonwealth Scientific and Industrial Research Organisation, Sydney, NSW 2145, Australia; Australian e-Health Research Centre, Commonwealth Scientific and Industrial Research Organisation, Sydney, NSW 2145, Australia; Department of Biomedical Sciences, Macquarie University, Sydney, NSW 2109, Australia; Health & Biosecurity Research Unit, Australian Centre for Disease Preparedness (ACDP), Commonwealth Scientific and Industrial Research Organisation, East Geelong, VIC 3219, Australia

## Abstract

**Motivation:**

Machine Learning approaches continue to be critical to the modelling of complex biological pathways, but many of the most-used models suffer from being uninterpretable, providing little insight into underlying mechanisms. Interpretable machine learning (IML) offers a pathway to bridge predictive modeling and biological understanding.

**Results:**

Here, we apply an IML framework combining TreeFARMS and Rashomon Set analysis to multi-omics data from a controlled ferret vaccination study as a case study. The dataset comprised transcriptomic, proteomic, lipidomic, and metabolomic profiles collected across ten time points from animals receiving different Inovio vaccine formulations. Using TreeFARMS, we generated sparse, interpretable decision trees optimized for accuracy and compactness, and explored their Rashomon Sets to identify stable and alternative molecular rules predictive of vaccination status. The resulting models outperformed the ensemble methods while producing concise if–then rules that directly linked measurable molecular features, such as AZGP1 expression and ketoleucine levels, to immune response patterns. This proof-of-concept study demonstrates that interpretable ML can capture biologically meaningful signatures of vaccine response without sacrificing predictive accuracy, providing a transparent and reproducible alternative to black-box approaches in multi-omics analysis.

**Availability and implementation:**

The datasets generated and/or analysed during the current study are available at: https://github.com/nehlehk/iml-ferret-vaccine/tree/main/data

## 1 Introduction

Machine learning (ML) has become a powerful tool in biological and clinical research, supporting tasks such as vaccine response prediction, biomarker discovery, and integration of heterogeneous omics data (Biswas and Chakrabarti 2020, Ng *et al*. 2023, Bravi 2024). This was emphasized by the recent SARS-CoV-2 pandemic, where ML was deployed to answer questions including genome classification (Singh *et al*. 2021), serological profiling (Rosado *et al*. 2021), prediction of viral–host protein interactions (Dey *et al*. 2020), and drug repurposing (Gawriljuk *et al*. 2021). However, many high-performing ML models, particularly those based on deep learning or ensemble approaches, function as black boxes offering limited insight into the biological mechanisms underlying their predictions. This lack of interpretability poses a major barrier for translational and clinical applications, where transparency and trust are essential.

Interpretable machine learning (IML) provides a pathway to address this challenge by producing models that are both accurate and explainable. In this study, we apply an IML framework to derive transparent, rule-based models from multi-omics data generated in a controlled ferret vaccination experiment. Whereas previous analyses of this dataset (Lee *et al*. 2026) emphasized feature ranking via ensemble models, we demonstrate that interpretable ML can achieve comparable accuracy while directly revealing the molecular logic of vaccine response. Our approach serves as a proof of concept illustrating how interpretable models can complement traditional black-box workflows by providing concise, actionable rules that connect measurable molecular features to vaccination outcomes.

In practice, decision-makers often require models that are not only accurate but also transparent and easy to interpret. Interpretable ML has a long history, most notably in decision trees and rule lists, which present predictions as simple if–then rules (Quinlan 2014, Yang *et al*. 2017, Angelino *et al*. 2018). However, classical greedy tree methods such as CART or C4.5 often produce unstable, suboptimal structures, while rule lists are limited to a single decision path. More recent work has introduced optimal sparse tree learners (Hu *et al*. 2019, Verwer and Zhang 2019, Aglin *et al*. 2020), but these methods still focus on selecting a single “best” model.

Beyond selecting a single “best” model, the Rashomon perspective (Fisher *et al*. 2019) recognizes that many models can achieve near-identical performance using different sets of variables. Exploring this model family reveals both stable rules, features consistently predictive across solutions, and alternative explanations, offering a richer view of biological heterogeneity. Application of this method to complex biological datasets provides the opportunity to extract biologically relevant rules that can help explain complex processes.

Here we have employed the TreeFARMS framework, coupled with Rashomon set exploration, to identify compact decision rules that distinguish vaccinated from unvaccinated ferrets. By exploring not only a single optimal model but also a family of near-optimal ones, our approach captures both stable and alternative molecular signatures associated with vaccine-induced immune responses. As a proof of concept, we have analyzed a ferret vaccine response dataset with the objective of identifying molecular features that differentiate the immune responses elicited by the different vaccination regimens. This dataset is comprised of a small number of wide samples (i.e. small-n, large-p), with which classical machine learning approaches typically struggle. This case study demonstrates the feasibility and utility of interpretable machine learning in complex multi-omics analyses, bridging the gap between predictive performance and biological insight.

## 2 Methods

**Figure 1:**
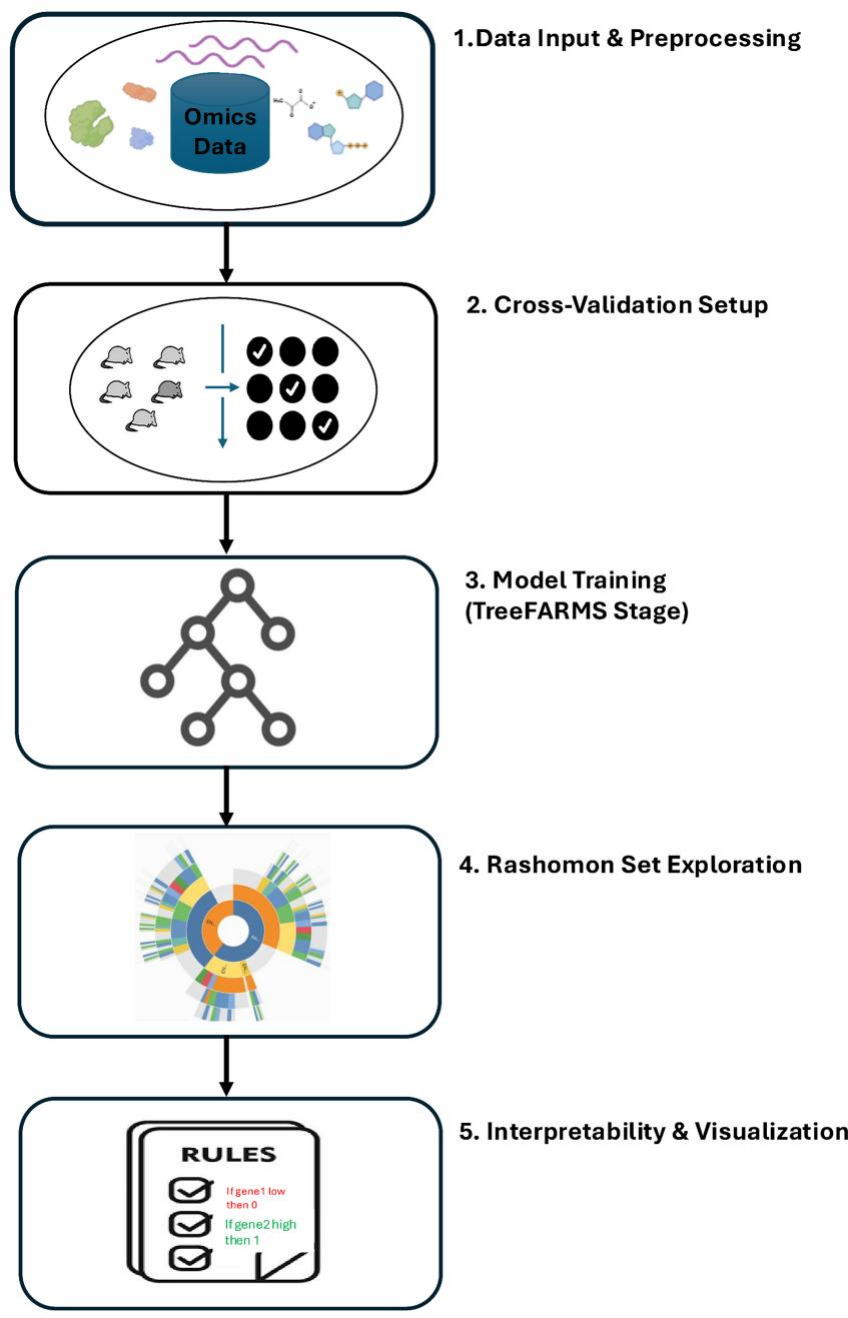
Schematic overview of the interpretable machine learning pipeline. (1) Data input and preprocessing (omics data: multi-layer biological datasets). (2) Cross-validation setup. (3) Model training using TreeFARMS (Tree-based Feature-Aggregated Rashomon Model Selection). (4) Rashomon set exploration. (5) Interpretability and visualization of rule-based outputs.

The workflow consisted of five stages: (1) data input and preprocessing, (2) cross-validation setup, (3) model training with TreeFARMS, (4) Rashomon set exploration, and (5) interpretability and visualization. A schematic overview of this workflow is shown in Fig. 1.

### 2.1 Dataset design

We analyzed data from a controlled ferret vaccination study involving sixteen adult, desexed male ferrets (*Mustela putorius furo*) that were randomly assigned to vaccinated and control groups. Vaccinated animals received two intramuscular doses of DNA vaccines of either INO-4800 (Wuhan-Hu-1 sequence) or INO-4802 (synthetic consensus sequence), administered on Day 0 (prime) and Day 28 (boost), while control ferrets received phosphate-buffered saline (PBS). The study followed animals for ten time points, including baseline and post-challenge stages, to capture longitudinal immune responses (Fig. S1, available at supplementary material  *Bioinformatics Advances* online). Multi-omics profiling was performed on blood and plasma samples, integrating transcriptomic, metabolomic, lipidomic, and proteomic measurements to characterize vaccine-induced changes across molecular layers (Lee *et al*. 2026).

For this work each timepoint was treated as an independent sample for modelling purposes to increase statistical power and mitigate the effect of repeated measures. Each sample was represented as a vector of multi-omics features. To ensure data quality, features with more than 20% missing values overall or more than 50% missing within a group were removed, followed by imputation, normalization, transformation, and scaling. This dataset enabled us to classify vaccinated vs. unvaccinated animals, and identify interpretable molecular signatures associated with vaccine protection (Fig. S1, available at supplementary material  *Bioinformatics Advances* online).

### 2.2 Model workflow

Machine learning analysis was performed using the TreeFARMS framework (Xin *et al*. 2022), which constructs families of accurate and reliable models through scalable optimal tree learning. The workflow was implemented in Python (TreeFARMS v1.0) and followed the two-stage process outlined in the original method.

To ensure generalizability, the data were split by animal using 5-fold stratified cross-validation, ensuring that all samples from a given ferret were contained entirely within a single fold. Features were preprocessed using the threshold guessing algorithm from the TreeFARMS package to identify informative cut points for each feature, balancing granularity and stability. Continuous features were discretized using the threshold-guessing algorithm implemented in the TreeFARMS package. Specifically, the function *compute_thresholds* evaluates each continuous variable and identifies informative cut points by examining feature distributions and class separation. This procedure balances granularity with stability, ensuring that only thresholds that meaningfully contribute to predictive discrimination are selected.

The resulting threshold set was calculated on the training fold and applied to the test data using the cut function, which converts each continuous feature into one or more binary indicator variables (e.g. feature ≤ threshold, feature > threshold). This transformation enables efficient mixed-integer optimization in downstream rule-based model training while preserving relevant structure in the original continuous variables.

Model training was performed using the GOSDT (Generalized Optimal Sparse Decision Trees) solver backend, which formulates decision tree learning as a global optimization problem. It minimizes a joint objective that balances classification error and tree complexity through a tunable regularization parameter (*λ* = 0.008). Each resulting model represents a compact, interpretable set of if–then rules describing relationships between molecular features and vaccination status.

For each training fold, TreeFARMS generated a collection of near-optimal trees within a Rashomon bound. This bound defines the set of all models whose loss is within 5% of the global optimum and an accuracy of 75% or greater on the test fold, ensuring inclusion of alternative high-performing but distinct rule sets. The collection of trees across all training folds is referred to as the Rashomon Set.

Model outputs, including rule structures, feature usage, and prediction paths, were automatically extracted and summarized to quantify rule stability and feature recurrence across folds. Stable features appearing in multiple near-optimal trees were considered biologically robust predictors of vaccine response.

To assess rule consistency and feature stability across near-optimal models, rule paths and feature occurrences were extracted from each TreeFARMS-generated tree. All if–then paths and their associated predictions were parsed from model outputs, and the frequency of each unique rule and feature was calculated across cross-validation folds. The resulting summaries quantified the recurrence of rules and features within the Rashomon Set. Features appearing consistently across multiple near-optimal trees were interpreted as robust molecular signatures predictive of vaccination status.

Five other machine learning methods were used for comparison: Random Forest, LassoNet, Decision Tree, LASSO and ANOVA with Logistic Regression. Table 1 summarizes the key parameters used to train all models in this study. These additional models were trained on the non-discretized training set using the same test/training fold-splits as TreeFARMS.

**Table 1:** Key model parameters.

Method	Key parameters
TreeFARMS	Reg = 0.008, rashomon_bound_multiplier = 0.05
Random Forest	500 tree, oob_score=True, random_state = 20
LASSO	L1 penalty, liblinear solver, max_iter = 2000, random_state = 20
Decision Tree	Max_depth = 5, random_state = 20
ANOVA + LogReg	SelectKBest(f_classif, *k* = 50) + L1 LogisticRegression, random_state = 20
LassoNet	LassoNetClassifierCV, random_state20 (internal CV lambda selection)

## 3 Results

### 3.1 TreeFARMS produces accurate and concise models

**Figure 2:**
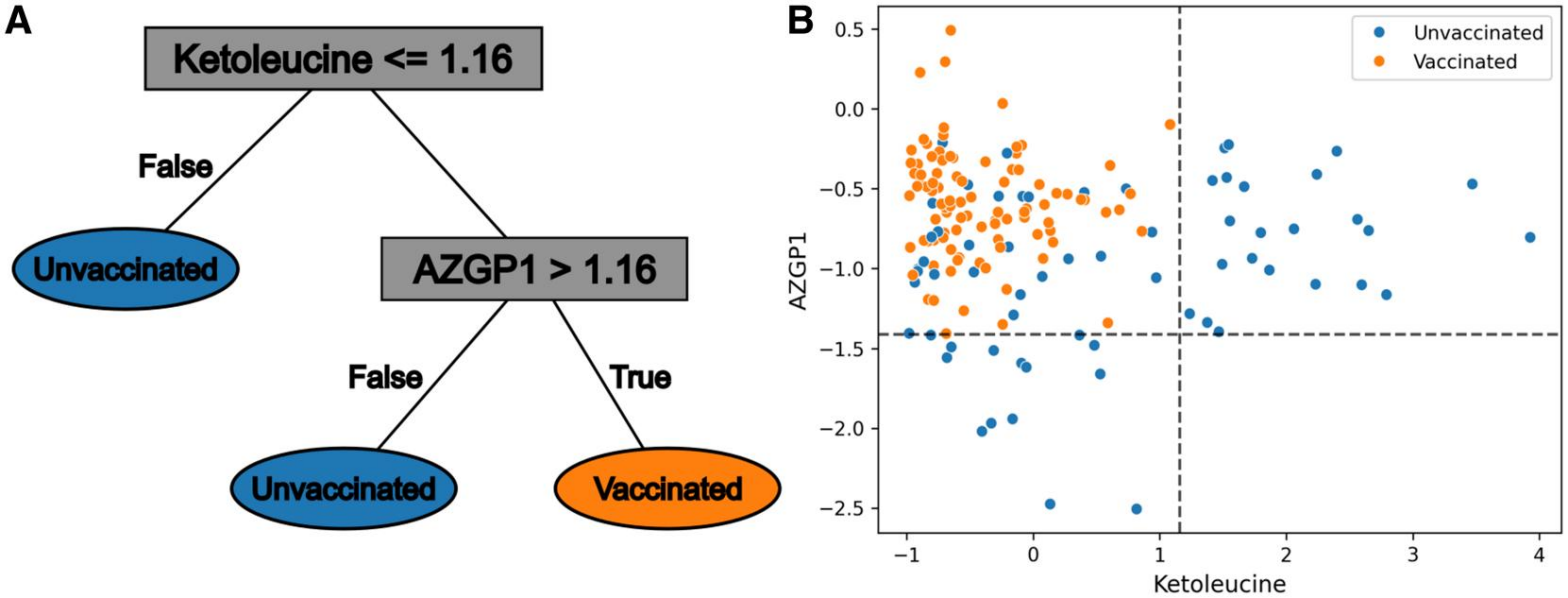
TreeFARMS builds highly interpretable models. The Rashomon set consists of multiple models consisting of simple if/else statements linking one or more features to the predicted class. (A) is an example of one such rule, linking the levels of the metabolite Ketoleucine and AZGP1 expression to vaccination status. (B) This rule clearly demarcates the boundary between vaccinated and unvaccinated samples as marked by the dashed lines.

The Rashomon Set developed by TreeFARMS achieved a mean accuracy in classifying vaccinated vs. control ferrets of 86.3% with a standard deviation of 8.4%. In total, the Rashomon Set contained 188 models each containing an average of 5 features organized in 2–4 concise if–then rules connecting one or more omics features to vaccination status. These rules represent a simple decision tree-like workflow for determining vaccination status and can be represented as such. One of the key rules that was overrepresented across training folds was the interaction between the levels of the metabolite Ketoleucine and expression of the AZGP1 gene. High levels of Ketoleucine indicated an unvaccinated sample. While low levels of Ketoleucine were only an indicator of vaccination if AZGP1 Expression was high. This relationship can be mapped as a simple decision tree (Fig. 2A) and is clearly apparent when plotting levels of the two features (Fig. 2B).

Exploration of the 188 near-optimal models within the Rashomon set built across all training folds revealed consistent biological signals across diverse decision structures. Features such as AZGP1, Ketoleucine, and WDR64 recurred in over 70% of high-performing trees, indicating robust, biologically stable predictors.

Table 2 summarizes the most recurrent features with many of them linked to key processes in immune modulation, metabolic adaptation and cytokine regulation. This overlap reinforces the validity of interpretable models for capturing essential biological signals while remaining fully explainable.

**Table 2:** Top 10 stable molecular features repeatedly selected across Rashomon models.

Feature Name	Omics Type	Recurrence (%)
AZGP1	Transcriptomic	100
Ketoleucine	Metabolomic	100
Unknown (14 987)	Transcriptomic	86
WDR64	Transcriptomic	75
Unknown (26 188)	Transcriptomic	63
EMILN2	Transcriptomic	49
NLRP3	Transcriptomic	40
PRDM16	Transcriptomic	33
CLDN1	Transcriptomic	31
2-Methoxyestrone	Metabolomic	9

### 3.2 TreeFARMS outperforms other models in both accuracy and simplicity

To contextualize the performance of TreeFARMS, we compared it to five commonly used baseline models: Random Forest, LASSO, Decision Trees, ANOVA + Logistic Regression and LassoNet. These models represent a selection of methods across Ensemble, Linear and Tree based approaches that are commonly used in biological analysis. Each model was trained on the non-discretized ferret samples using the same stratified test/train folds as the TreeFARMS pipeline. Table 3 summarizes the performance of all models averaged across the test sets.

**Table 3:** Performance of the different predictive models.

Method	Accuracy (*SD*)	Features selected	Model type
TreeFARMS	86.3 (8.4)	5	Rashomon Set
Random Forest	75.3 (16.1)	1338	Ensemble
LASSO	71.2 (19.9)	52	Linear
Decision Tree	73.0 (7.7)	7	Tree
ANOVA + LogReg	63.8 (21.5)	16	Linear
LassoNet	74.2 (24.1)	1293	Linear

**Figure 3:**
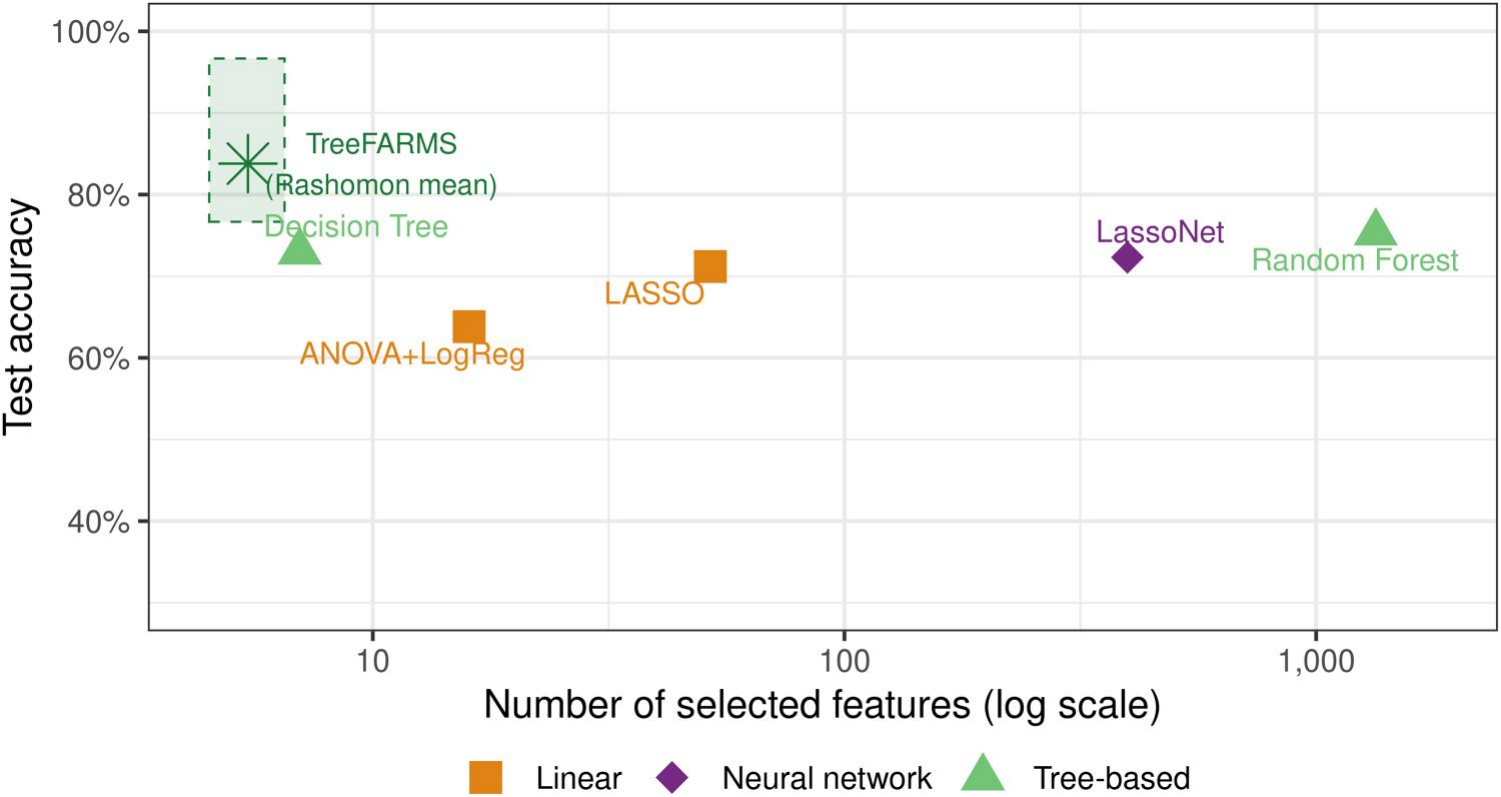
TreeFARMS models are simpler and more accurate. Comparison of model complexity (measured by the numbers of features chosen for the final model) and accuracy averaged across all training folds. Models are colored by their type. Note that the TreeFARMS result is the average across the entire Rashomon Set, which is contained within the surrounding shaded area.

TreeFARMS outperformed all models, showing the highest accuracy with the smallest number of features. Random Forest produced the second highest accuracy (75.3 ± 16.1%) but required 1338 features compared to the Rashomon Set’s average of 5. A Decision Tree approach was possible with only 7 features but yielded a much worse performance (73.0 ± 7.7%). Fig. 3 demonstrates the differences in accuracy and feature selection of the 6 models, clearly showing that TreeFARMS outperforms the other models in both accuracy and simplicity (Table 3).

We next examined whether the different algorithms converged on similar biomarkers of vaccination response. Each model provides a measure of feature importance and these were averaged across all training folds to identify the 10 most informative features for each model (Table S1, available at supplementary material  *Bioinformatics Advances* online). Table 4 summarizes characteristics of the models including overlap with the top TreeFARMS features and the types of features. Genes were the most represented feature type with only a few metabolites present in the Top 10 and no lipids. The exception was LassoNet which predominantly favored metabolites. Ketoleucine, a metabolite linked to inflammation and energy metabolism was identified as informative by all models and AZGP1 expression prioritized by all models except Random Forest.

**Table 4:** Comparison of recurrent features across models.

	Overlap with TreeFARMS	No. of genes	No. of metabolites	No. of lipids
TreeFARMS	–	8	2	0
Random Forest	5	8	2	0
Decision Tree	4	7	3	0
LASSO	3	6	4	0
ANOVA + LogReg	4	8	2	0
LassoNet	2	3	7	0

A key challenge in biology is small-n/large-p datasets, that is datasets with a small number of samples but a large feature-set such as in this study. While the individual time-points were treated as separate samples for modelling purposes, the effective sample number in this study remains 16. With such a small sample size, the influence of outliers can be disproportionate. Investigation of the distribution of important features across the individual ferrets revealed potential outliers in 2 animals based on their Ketoleucine levels (Fig. S2, available at supplementary material  *Bioinformatics Advances* online). The ferret vaccinated samples from Ferret 9 showed a higher level of Ketoleucine compared to the cohort average while the unvaccinated control Ferret 12 showed a much lower level compared to the others. Given the importance of Ketoleucine to all models, this could be indicative of outliers skewing the results.

To test the impact of these two outliers, the modelling workflows were re-run removing all samples associated with these two ferrets. When removed, TreeFARMS accuracy across training folds increased to 88.7 ± 7.6% and the Rashomon Set grew to 369 trees, indicating an increased ability to model vaccination status. LASSO and LassoNet also showed small increases in accuracy after removal of the outliers while all other models showed a decrease (Table 5). These changes in accuracy were also accompanied by a change in important features (Table S2). There were 4 new features in the Top 10 for TreeFARMS. For the other models it ranged from 3 new in the Random Forest model to 10 new for LassoNet. Interestingly, AZGP1 which was consistently rated as a highly informative feature across all models before outlier exclusion was only retained by the ANOVA + LogReg. The reduction in importance of AZGP1 suggests that this feature was specifically chosen to assist in modelling the outlier samples.

**Table 5:** Model accuracy with and without outliers.

	Accuracy with outliers (*SD*)	Accuracy without outliers (*SD*)
TreeFARMS	86.3 (8.4)	**88.7 (7.6)**
Random Forest	75.3 (16.1)	**71.3 (22.2)**
LASSO	71.2 (19.9)	**72.0 (16.6)**
Decision Tree	73.0 (7.7)	**60.0 (23.5)**
ANOVA + LogReg	63.8 (21.5)	**62.0 (13.0)**
LassoNet	74.2 (24.1)	**75.7 (16.2)**

## 4 Discussion

This case study demonstrates the practical value of interpretable machine learning for understanding vaccine response mechanisms in complex multi-omics datasets. Using TreeFARMS with Rashomon analysis, we re-examined the ferret vaccination data originally analyzed by Lee *et al.* (2026) showing that interpretability and predictive accuracy can be achieved simultaneously. While the most informative features were common across all model approaches, TreeFARMS and Rashomon Set analysis presented them in a clear way that clearly demonstrated relationships and interactions between features allowing for easy comprehension and down-stream hypothesis development.

TreeFARMS produced compact, easily readable models that achieved cross-validated accuracies averaging 86%, outperforming other commonly used machine learning approaches. While additional ensemble approaches such as XGBoost or gradient boosting could be considered, Random Forest serves as a representative black-box baseline, offering a robust comparison for assessing the trade-off between interpretability and accuracy.

The stable features summarized in Table S1 highlight molecular processes central to vaccine-induced immunity. For instance, AZGP1 and Ketoleucine point to zinc-associated signaling and branched-chain amino acid metabolism respectively, both key regulators of immune activation and metabolic adaptation. The recurrence of CLEC4D and NLRP3 underscores the involvement of innate immune sensing and inflammasome signaling, while VCAN and CLDN1 reflect extracellular and barrier components modulating immune interactions. Together, these reproducible features illustrate how interpretable models not only match black-box accuracy but also pinpoint biologically coherent pathways.

The extracted rules, such as low Ketoleucine and high AZGP1 predicting vaccination, provide a direct link between measurable molecular features and vaccination status. These features are consistent with broader evidence that branched-chain amino-acid metabolism and zinc-associated glycoproteins participate in immune and inflammatory regulation, although their precise roles in vaccine responses remain to be clarified (Kelly and Pearce 2020, Ling *et al*. 2023). These interpretable ML findings complement the statistical analyses performed in the original study. In that work, differential expression analysis identified several vaccination-associated genes.

Beyond identifying a single best model, Rashomon exploration revealed multiple, equally accurate trees containing overlapping but distinct rule combinations. This diversity reflects the biological redundancy of immune mechanisms, where different molecular pathways can yield comparable protective responses. Stable rules, such as those involving Ketoleucine, AZGP1, and CLEC4D, appeared across most high-performing trees, confirming their robustness and biological relevance.

Compared with ensemble approaches that provide only aggregate feature importance scores, TreeFARMS explicitly enumerates all near-optimal solutions, allowing direct comparison of interpretable hypotheses. This capacity to visualize both stable and alternative rules enhances scientific transparency and supports responsible AI principles in genomic research.

A key advantage of the TreeFARMS framework is its use of the GOSDT solver as the underlying optimization engine. In contrast to classical greedy approaches such as CART or C4.5, which make a sequence of locally optimal split decisions, GOSDT performs a global search over the decision-tree space using dynamic programming and branch-and-bound. This procedure provides a mathematical guarantee that the resulting tree is globally optimal under the specified objective, jointly minimizing classification error and tree complexity controlled by the regularization term *λ*. As a result, the interpretable models produced by TreeFARMS achieve the best attainable accuracy–simplicity trade-off within the sparse tree family.

GOSDT further maintains tight upper bounds on the potential performance of partially explored trees, allowing it to prune entire regions of the search space that cannot fall within the user-defined Rashomon bound. This makes exhaustive searches computationally feasible while ensuring that no high-performing alternative trees are missed.

Crucially, this optimization structure enables complete enumeration of the Rashomon set through TreeFARMS. Prior methods typically rely on repeated sampling, perturbation, or bootstrap procedures to approximate near-optimal models, often producing incomplete or biased subsets. In contrast, TreeFARMS guarantees discovery of all sparse decision trees whose loss lies within the Rashomon bound of the global optimum (Xin *et al*. 2022). This complete enumeration provides a uniquely comprehensive view of model uncertainty, allowing us to assess rule stability, feature recurrence, and biological plausibility across all 188 near-optimal models rather than relying on sampled approximations.

In our workflow, “optimality” occurs at two different levels. First, GOSDT provides a globally optimal sparse decision tree by minimizing a regularized objective that combines classification error and tree complexity. This optimality is unique to GOSDT and cannot be achieved by greedy tree algorithms such as CART. Second, TreeFARMS explores the Rashomon set, defined as all trees whose classification error lies within 5% of the minimum attainable error. This near-optimality is a property of the solution landscape rather than the optimization objective. Thus, the Rashomon set includes many high-performing models, whereas the GOSDT-optimal tree is a single, provably optimal solution to the regularized objective.

Another notable strength of the TreeFARMS framework is its suitability for small-sample, high-feature datasets: rather than overfitting, it produces compact rule sets that generalize well even when the number of features vastly exceeds the number of individuals. While in this study the individual time-points were treated as separate samples for modelling purposes, the effective *n* remained as 16. In such small sample sizes, the impact of outliers can be exaggerated. In this study, two ferrets were identified as potential outliers based on PCA clustering. While removal of them did improve the TreeFARMS model, the largest impact was on the selected features. AZGP1 expression, which had been selected as the second most important feature, was no longer in the final model. This is because AZGP1 was used to properly classify these outlier samples. While other models hide how this relationship works exactly, the rules from TreeFARMS show it explicitly with AZGP1 expression being directly linked to Ketoleucine levels (Fig. 2A) highlighting the interpretability of the Rashomon set approach.

By embedding interpretability within the modelling process, rather than applying it post hoc, our framework allows decision-makers and experimentalists to trace each prediction to specific molecular conditions. Such transparency strengthens trust, facilitates biological validation, and enables integration of machine learning results into experimental design and public-health decision support through the identification of biomarkers and signatures for key clinical traits such as disease severity, immune response and therapeutic efficacy.

Future work will extend this approach to larger cohorts and additional omics layers, assessing how Rashomon-derived rule sets generalize across vaccine platforms or host species as well as how they handle time-series data. Integrating interpretable rules with mechanistic or causal models could further validate their biological significance and support explainable, reproducible analytics for next-generation vaccine studies.

## Supplementary Material

vbag167_Supplementary_Data

## Data Availability

The datasets generated and/or analysed during the current study are available at: https://github.com/nehlehk/iml-ferret-vaccine/tree/main/data
