# Supplementary material for: Interpretable machine learning for low-sample multi-omics: a case study of ferret vaccine response
Source: Bioinform Adv. 2026 Jun 12;6(1):vbag167. doi: 10.1093/bioadv/vbag167 (PMC13316433; doi:10.1093/bioadv/vbag167)
Supplement: vbag167_Supplementary_Data [file vbag167_supplementary_data.docx]

**Supplementary Data**


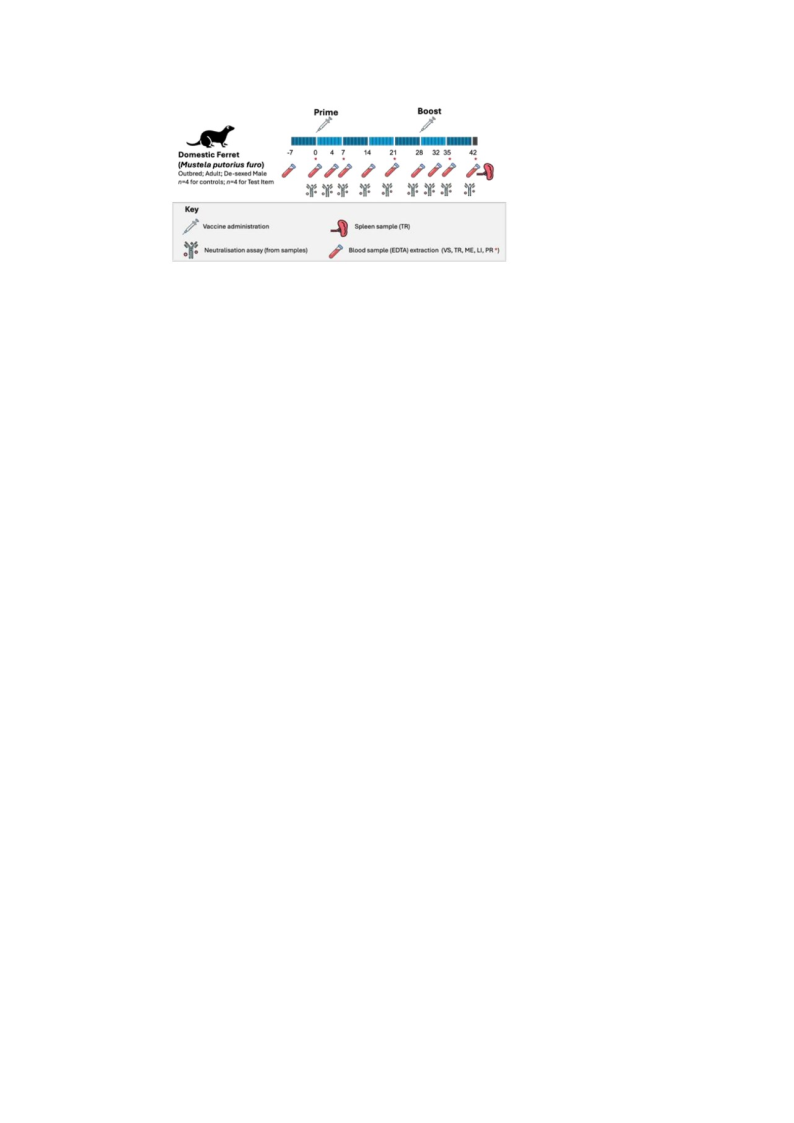


**Figure 2.** Experimental design of the ferret vaccination study.
Four treatment groups of ferrets received prime and boost vaccinations on Days 0 and 28. Multi-omics samples (transcriptomics, metabolomics, lipidomics and proteomics) were collected from blood and spleen at the indicated time points, following the workflow developed by the broader study. In this work, we analyse the integrated multi-omics data generated at the endpoint sampling time point only. Abbreviations: PBS = phosphate-buffered saline; TR = transcriptomics; ME = metabolomics; LI = lipidomics; PR = proteomics.

**Figure S2:** Ketoleucine levels of potential outlier samples

The average Ketoleucine levels were calculated across all vaccinated and unvaccinated samples for Ferret 12 and 9 and compared to the averages of the rest of the cohort. Ferret 9 showed a high base level of Ketoeleucine across vaccinated samples while the unvaccinated samples of Ferret 12 showed a low Ketoleucine level both in contrast with the averages across the other ferrets indicating potential outliers. Note Ferret 12 was an unvaccinated control and does not contain any vaccinated samples.

**Table S1: Top 10 features selected across all tested models**

| **TreeFARMS** | **Random Forest** | **LASSO** | **Decision Tree** | **ANOVA + LogReg** | **LassoNet** |
| --- | --- | --- | --- | --- | --- |
| AZGP1 | NLRP3 | TCN1 | EMILIN2 | CLDN1 | L-Tryptophan |
| Ketoleucine | EMILIN2 | Ketoleucine | Ketoleucine | Ketoleucine | CSTA |
| Unknown (14987) | CLDN1 | Unknown (14987) | NLRP3 | HGF | Ketoleucine |
| WDR64 | CXCL14 | Allylmalonic acid | Unknown (14987) | ITM2C | L-Lysine |
| Unknown (26188) | TLR5 | Unknown (24388) | ST6GALNAC1 | Unknown (25034) | Unknown (10655) |
| EMILIN2 | Ketoleucine | MKRN3 | WBP2NL | Unknown (14987) | Allylmalonic acid |
| NLRP3 | D-Glyceric acid | Unknown (26895) | PRDM16 | AZGP1 | WDR64 |
| PRDM16 | HSD17B11 | L-Tryptophan | L-Norleucine | Unknown (10655) | D-Glyceric acid |
| CLDN1 | Unknown (14987) | WDR64 | Allylmalonic acid | Unknown (27308) | Guanosine |
| 2-Methoxyestrone | B3GALT2 | L-Alanine | Unknown (9853) | D-Glyceric Acid | Guanidinosuccinic acid |

**Table S2: Top 10 features selected across all tested models with outlier samples removed**

| **TreeFARMS** | **Random Forest** | **LASSO** | **Decision Tree** | **ANOVA + LogReg** | **LassoNet** |
| --- | --- | --- | --- | --- | --- |
| Ketoleucine | TLR5 | Ketoleucine | Ketoleucine | Ketoleucine | **IL1R2** |
| Unknown (14987) | NLRP3 | TCN1 | **HSDB17B11** | **TLR5** | **Unknown (26672)** |
| **CXCL14** | EMILIN2 | **Unknown (10655)** | EMILIN2 | **SCN11A** | **LRRC55** |
| **COLQ** | Ketoleucine | Allylmalonic acid | **Unknown (2125)** | **HSD17B11** | **CD300E** |
| EMILIN2 | CXCL14 | WDR64 | **COLQ** | Unknown (14987) | **CEACAM18** |
| NLRP3 | **COLQ** | **Unknown (2911)** | **Unknown (10655)** | **COLQ** | **Unknown (26392)** |
| WDR64 | HSD17B11 | **2-Hydroxyhexadecanoic acid** | **2-Deoxycytidine 5 diphosphate** | AZGP1 | **Unknown (29944)** |
| **D Lyxose** | CLDN1 | **WBP2NL** | WBP2NL | **NLRP3** | **Cytosine** |
| Unknown (26188) | **ANXA1** | Unknown (14987) | Unknown (14987) | **WDR64** | **PLVAP** |
| **TYMS** | **2-Methoxyestrone** | **Glutaric acid** | **Glutaric acid** | CLDN1 | **TRBV12-3** |

* Bold indicates new features within the top 10
